# Interplay Between Ribosomal Gene Deficiency and Calorie Restriction in Shaping Yeast Biosynthetic Capacity

**DOI:** 10.3390/cells14231901

**Published:** 2025-12-01

**Authors:** Roman Maslanka, Renata Zadrag-Tecza

**Affiliations:** Faculty of Biology, Nature Protection, and Sustainable Development, University of Rzeszów, 35-601 Rzeszów, Poland; rmaslanka@ur.edu.pl

**Keywords:** ribosomes, cell size, biosynthetic potential, calorie restriction

## Abstract

**Highlights:**

**What are the main findings?**

- Low biosynthetic activity results in slower growth, smaller cell size, and an extended cell cycle, primarily affecting the G1 phase.
- In the Δ*rpl20a* strain, which exhibits reduced biosynthetic activity, calorie restriction did not lead to any additional significant changes in cell growth rate or biosynthetic activity.

**What are the implications of the main findings?**

- These results suggest there is a minimum biosynthetic threshold necessary to maintain cellular physiological fitness.
- The impact of calorie restriction on physiological fitness and proliferative potential depends on the baseline level of cellular biosynthetic activity.

**Abstract:**

Biosynthetic capacity, particularly in protein production, significantly influences cells’ physiological efficiency and their ability to proliferate. Numerous studies have suggested that reducing protein synthesis can extend lifespan and improve indicators of cellular efficiency. However, the precise mechanism behind this phenomenon remains unclear. This study employed a model combining two methods to reduce biosynthetic activity—the deletion of genes encoding ribosomal proteins (e.g., *RPL20A*, *RPL20B*, and *RPS6B*) and calorie restriction—to analyse the relationships among biosynthetic capacity, cell size, and physiological efficiency. The results indicate that, under calorie restriction, parameters such as growth rate, cell size, protein content, metabolic activity, and glucose utilisation decrease significantly only in the wild-type strain. Conversely, yeast strains with deleted ribosomal protein genes—particularly Δ*rpl20a*, which has the lowest biosynthetic capacity—exhibit a marked reduction in biosynthetic capacity under optimal conditions and no additional limiting effect from calorie restriction. These findings suggest the existence of a minimal threshold of biosynthetic capacity required to maintain a cell’s physiological efficiency. Thus, factors that reduce biosynthetic efficiency only have a noticeable effect on cells with biosynthetic activity levels above the minimal threshold.

## 1. Introduction

The physiological efficiency of cells, typically measured by their ability to proliferate, results from the coordination and interaction of metabolism, growth, and cell division. Changes in central carbon metabolism noted during particular phases of cell cycle or individual stages of cell life provide appropriate levels of biosynthesis and energy availability [1,2]. The level of biosynthesis, closely related to protein synthesis, directly affects cell growth and proliferation. Therefore, it must be strictly regulated and adapted to environmental conditions, including stress conditions, during which a reduction in protein synthesis may prevent the impairment of cell proteostasis by the accumulation of damaged proteins [3,4,5]. The production of new proteins requires, among other things, amino-acyl-tRNAs and energy precursors, as well as ribosomes, which serve as the molecular machinery. Ribosomes in eukaryotic cells are composed of four types of ribosomal RNA (rRNA) and 79 ribosomal proteins (RP), forming two subunits [6]. The small 40S ribosomal subunit contains one kind of ribosomal RNA, i.e., 18S rRNA, and 33 different ribosomal proteins. In contrast, the large 60S ribosomal subunit contains three rRNAs (5S, 5.8S, 25S) and 46 ribosomal proteins [7]. For a long time, it was assumed that ribosomes were homogeneous in both structure and function. This view is still evolving significantly in light of data indicating the heterogeneity of these structures across various groups of organisms [8,9,10,11]. At least several ways of generating ribosomal heterogeneity have been proposed, including the substitution of ribosomal protein paralogs [12], the differential ribosomal protein stoichiometry [13], post-translational modification of ribosomal proteins [14] or rRNA modifications [15]. It is believed that differences in the composition of the ribosome population may constitute a mechanism regulating mRNA translation, among others, because different groups of ribosomes can be adapted to translate specific groups of mRNAs [16]. The presence of ribosomal protein paralogs has been observed in many organisms, including the yeasts *Saccharomyces cerevisiae* and *Schizosaccharomyces pombe* [17,18], *Drosophila melanogaster* [19], and human cells [20]. As a result of genome duplication, 59 of the 79 ribosomal proteins in the yeast *S. cerevisiae* exist in the form of paralogs [21]. Despite the high sequence similarity between paralogs, removing one paralog from a pair often results in a distinct phenotype, suggesting functional diversity [17,22]. Protein synthesis is considered one of the most energy-consuming cellular processes [23]; thus, modifications that reduce mRNA translation allow the redirection of key energy resources, among others, towards processes involved in repairing damage and maintaining cell efficiency [24]. This concept is supported by research showing that reducing protein synthesis by depleting ribosomal proteins and translation factors increases stress resistance and extends lifespan [25]. This is also supported by studies using pharmacological inhibition of protein translation. The usage of low doses of cycloheximide, a protein synthesis inhibitor, reduces the ageing-related features in human cells and extends the lifespan of *C. elegans* [26]. Similarly, diazaborine, which reduces levels of 60S ribosomal proteins and inhibits ribosome biogenesis and maturation, significantly increases the replicative lifespan of budding yeast [27].

In recent years, various mechanisms have been proposed to explain how the rate of protein synthesis may influence proliferation. The rapid rate of protein synthesis increases the risk of damaged protein formation due to disturbances in transcription, translation, or misfolding. Their accumulation, in the form of aggregates in the cell, is considered one of the key causes of stopping cell proliferation and, consequently, ageing [28]. Therefore, slowing down translation may act as a kind of protection against the production of excessive amounts of misfolded proteins and overloading the protein quality control system [29]. Numerous studies have demonstrated that long-term restriction of protein synthesis markedly enhances both lifespan and various parameters related to the physiological efficiency of cells, although protein synthesis facilitates growth and proliferation (reviewed in [30]. This is the so-called protein synthesis paradox [31], which emphasises the importance of explaining how protein synthesis controls the physiological efficiency of cells and their capacity to proliferate.

Considering the above arguments, we set out to examine the physiological effects of reducing biosynthetic efficiency in cells, both at the individual and population levels. The experimental system used reduced biosynthetic activity in cells by depriving selected ribosomal proteins of their association with either the large or the small ribosomal subunit, as well as through calorie restriction (CR). The results indicate that, regardless of the approach used to reduce cellular biosynthesis—whether genetic or environmental—the significant outcome is a reduction in cell size and growth rate, which may help explain the increased proliferative capacity of cells in conditions of reduced protein synthesis. Furthermore, the CR effect is not observed in cells with low biosynthetic efficiency, such as those of yeast strains with particular deletions of RP genes, suggesting the existence of a lower limit to a cell’s biosynthetic capacity required for its functionality.

## 2. Materials and Methods

### 2.1. Chemicals

6-(N-(7-nitrobenz-2-oxa-1,3-diazol-4-yl) amino)-6-deoxyglucose (6-NBDG) and FUN^®^1 Cell Stain were from Molecular Probes (Eugene, OR, USA). BacTiter-Glo™ Microbial Cell Viability was from Promega (Madison, WI, USA). Coomassie Protein Assay Reagent (Thermo Scientific, Rockford, IL, USA). Alpha-Factor Mating Pheromone was purchased from Zymo Research (Irvine, CA, USA). All other reagents were purchased from Sigma-Aldrich (Poznan, Poland). Components of culture media were from BD Difco (Becton Dickinson and Company, Spark, MD, USA) except for glucose (POCH, Gliwice, Poland).

### 2.2. Yeast Strains and Growth Conditions

The following yeast strains were used: wild-type (WT) BY4741 (*MATa his3*Δ1; *leu2*Δ0; *met15*Δ0; *ura3*Δ0) and three mutant strains isogenic to BY4741: Δ*rpl20a* (BY4741; *MATa his3*Δ1; *leu2*Δ0; *met15*Δ0; *ura3*Δ0; YMR242c::kanMX4); Δ*rpl20b* (BY4741; MATa; *his3*Δ1; *leu2*Δ0; *met15*Δ0; *ura3*Δ0; YOR312c::kanMX4); and Δ*rps6b* (BY4741; *MATa*; *his3*Δ1; *leu2*Δ0; *met15*Δ0; *ura3*Δ0; YBR181c::kanMX4) (EUROSCARF, Scientific Research and Development GmbH, Oberursel, Germany). Yeast was grown in the liquid YP medium (1% Yeast Extract, 1% Yeast Bacto-Peptone) with different glucose concentrations (0.5% and 2%) on a rotary shaker at 150 rpm and 28 °C.

### 2.3. Determination of Cell Growth and Generation Time

The growth of yeast cells was analysed in the liquid medium. Yeast cultures were cultivated for 24 h in a shaking incubator Titramax 1000 (Heidolph Instruments GmbH & CO. KG, Schwabach, Germany) at 1200 rpm and 28 °C. The growth was monitored turbidimetrically at λ = 600 nm using an Anthos 2010 type 17 550 microplate reader (Anthos Labtec Instruments, Salzburg, Austria). Measurements were performed at 1 h intervals for 12 h and after 24 h of cultivation. The results are presented as a growth curve during 12 h and as OD values after 24 h. The generation time of cells in the population and the growth rate were calculated from the exponential phase of growth using appropriate formulas [32]. Cell density (number of cells per mL) was determined using a Malassez chamber (BRAND GmbH, Wertheim, Germany).

### 2.4. Estimation of Cell Size in the Population

The mean cell size in the population was estimated from microscopic images captured with an Olympus BX-51 microscope equipped with a DP-72 digital camera (Olympus, Tokyo, Japan). The diameter of the cell was measured using cellSens Dimension software 4.2.1 (Olympus, Tokyo, Japan). Cell diameter was measured in two perpendicular planes for each cell, and the mean value was used for calculations. The data represent the mean diameter calculated from individual cell measurements from two separate experiments. For each yeast strain cultured in a medium with 2% and 0.5% glucose concentrations, at least 200 cells were counted. Cell size was also presented as a violin plot. This type of plot provides a visual representation of the distribution of the analysed trait within the population.

### 2.5. Analysis of the Duration and Changes in Cell Size During the First Cell Cycle

The selected parameters of the first cell cycle were determined for cells cultured on agar plates using a micromanipulator and a Nikon Eclipse E200 microscope (Nikon, Tokyo, Japan) equipped with a SONY SSP-DC50AP digital camera (Sony Corporation, Tokyo, Japan). Briefly, one-microliter aliquots of overnight yeast cultures grown in YPD liquid medium were spotted onto YPD plates containing solid medium. For each experiment, single cells were micromanipulated to the appointed area, and the first daughters were chosen as the starting cells. The cells were observed throughout the cell cycle, and microscopic images were taken at designated time points. Cell size was measured at the start of the procedure, at the end of the G1 phase (when the first bud appeared), and at the end of the cycle (after bud separation). Measurements of 1st cell cycle duration include the time of the whole cell cycle, but also a separate determination of the time spent in the G1 phase, and the sum of the time spent in the S, G2, and M phases. Cell size was measured using the Micro Image 3.0 (Olympus, Tokyo, Japan) programme. For each cell, the diameter was measured in two perpendicular planes. The data represent the mean values obtained for individual cells from two separate experiments. The obtained values were also used to calculate the rate of increase in cell size during the cell cycle.

### 2.6. Analysis of Changes in Cell Size During the Cell Cycle Arrest in the G1 Phase

Cell suspension from the exponential phase was gently sonicated (30 s with an ultrasonic homogeniser, Sonic Ruptor 250, Omni International, Kennesaw, GA, USA) to separate mature buds from mother cells. Then, the virgin cells (buds) and cells with no more than 2–3 reproduction cycles were isolated by centrifugation (1000× *g*, 2 min). The bud and young cell suspension (10^6^ cells/mL) was spun down, re-suspended in fresh YPD medium, and treated with pheromone α (Zymo Research, Irvine, CA, USA) at a final concentration of 5 μM. The cell suspensions were incubated on a shaker (150 rpm, 30 °C) for selected time intervals (2, 4, and 6 h). After appropriate time intervals, the cells were centrifuged, and microscopic images were captured with an Olympus BX-51 microscope equipped with a DP-72 digital camera (Olympus, Tokyo, Japan). Due to changes in cell shape during incubation with pheromone, cell size was measured as both cell diameter and cell area. Both parameters were estimated using cellSens Dimension software. The obtained values were also used to calculate the rate of increase in cell size when cells were arrested in G1.

### 2.7. Determination of Cell Viability

Cell viability was determined by staining with PI (propidium iodide) and FDA (fluorescein diacetate) as described previously [33]. PI gets into dead cells, while FDA passively crosses the cell membrane and needs to be hydrolysed by intracellular esterases to the fluorescent product fluorescein. Dead cells (PI-positive) are red-fluorescent; in turn, live cells (FDA-positive) are green-fluorescent. FDA/PI fluorescence was examined from at least 100 cells in one biological replicate under a fluorescence microscope at λex = 480 nm. Viable cells were green fluorescent, and dead cells (PI-positive cells) were red fluorescent. The data represent the mean values obtained from three separate experiments.

### 2.8. Assessment of Cell Metabolic Activity

The relative metabolic activity of yeast cells, which can be treated as an equivalent of cell vitality, was determined with FUN^®^1 according to the manufacturer’s protocol (Molecular Probes) with the modification described by Kwolek-Mirek and Zadrag-Tecza [33]. FUN1 intracellularly is converted to a fluorescent product, allowing distinction among metabolically active, metabolically weakened, and dead cells. Cells with high metabolic activity contain cylindrical, red-fluorescent structures in the vacuoles; cells with low metabolic activity display only diffuse green fluorescence within the cytoplasm without fluorescent vacuolar inclusions. Incubation with 0.5 μM FUN 1 was conducted for 15 min in the dark at 28 °C. The fluorescence images of cells were captured with an Olympus BX-51 microscope equipped with the DP-72 digital camera (Olympus, Tokyo, Japan). Metabolically active and inactive cells were examined from at least 150 cells in one biological replicate. The data represent the mean values obtained from three separate experiments. Metabolically active cells contain red-fluorescent structures in their vacuoles (with or without green cytoplasmic fluorescence). In contrast, cells with no metabolic activity exhibit extremely bright, diffuse green cytoplasmic fluorescence without red fluorescent intravacuolar inclusions. The fluorescence of the cell suspension was also measured using a Tecan Infinite 200 microplate reader (Tecan Group Ltd., Männedorf, Switzerland) at λex = 480 nm and λem = 500–650 nm. Cellular metabolic activity was expressed as the ratio of red (λ = 575 nm) to green (λ = 535 nm) fluorescence.

### 2.9. Estimation of Relative RNA Level

Relative RNA content was determined according to the method using acridine orange (3,6-dimethylaminopyridine) described by Darzynkiewicz et al. [34] with modifications described by Zadrag-Tecza et al. [35]. Yeast cells from the exponential phase of growth were adjusted to a density of 1 × 10^8^ cells/mL and collected by centrifugation (2 min, 7000 rpm). The cells were fixed for 30 min in 70% cold ethanol. They were then washed twice with cold sterile phosphate-buffered saline (PBS) and suspended in fresh buffer to maintain the initial density. The cell suspension was mixed with permeabilisation buffer (0.1% Triton X-100, 80 mM HCl, and 150 mM NaCl) at a 1:2 ratio and incubated on ice for 2 min. Afterwards, the acridine orange solution (6 μg/mL) was added and incubated at a low temperature for 10 min. Microscopic observations of cells were performed using an Olympus BX-51 epifluorescence microscope equipped with a DP-72 digital camera (Olympus, Tokyo, Japan). The fluorescence images of cells were captured and analysed with the cellSens Dimension software 4.21 (Olympus, Tokyo, Japan). at λex = 488 nm and λem = 650 nm. To compare fluorescence signal intensity across the analysed yeast strains, images were acquired under the same conditions (lamp power and exposure time). The relative value of RNA was measured from red fluorescence after separating the multichannel images into individual colour channels. The relative value of RNA was defined as the red fluorescence intensity (the sum of the signal from pixels within the cell area) divided by the measured cell size. Analysis was performed on at least 150 cells for each strain and biological replicate. The quantitative results are presented as the mean value from two independent experiments for each cell.

### 2.10. Determination of Protein Content in the Yeast Cell

For extraction, 5 × 10^8^ yeast cells from the exponential-phase culture were used. Cells were centrifuged, washed twice with cold sterile water, and suspended in cold homogenization buffer (20 mM phosphate buffer with pH 6.8, containing 1 mM EDTA and 1 mM PMSF). The cells were disrupted with 0.5 mm glass beads, vortexed for 7 cycles of 30 s with cooling intervals on ice, and then centrifuged (14,000× *g*, 15 min, 4 °C). Supernatants were transferred to new tubes and immediately frozen at −80 °C. Protein concentration in the supernatant was determined using the Bradford method. The absorbance of samples was measured after 10 min of incubation with Coomassie Protein Assay Reagent (Thermo Scientific, Waltham, MA, USA) at room temperature using a Tecan Infinite 200 microplate reader (Tecan Group Ltd., Männedorf, Switzerland) at λ = 595 nm. The data were expressed as protein content per single cell. The data represent the mean values obtained from three separate experiments.

### 2.11. Assessment of the Cellular ATP Content

The level of ATP in yeast cells was determined with BacTiter-Glo™ Microbial Cell Viability Assay according to the manufacturer’s protocol (Promega, Madison, WI, USA) with its modifications. Cells from the exponential phase of growth were suspended in a 100 mM phosphate buffer with pH 7.0, containing 0.1% glucose and 1 mM EDTA. A sample of the cell suspension at a density of 10^6^ cells/mL was used for determination. The luminescent signal, proportional to the amount of ATP, was recorded using the Tecan Infinite 200 microplate reader (Tecan Group Ltd., Männedorf, Switzerland) after an appropriate time (until the luminescence signal obtained a stable level).

### 2.12. Measurement of Glucose Uptake

The glucose uptake rate was determined by using 6-NDBG, a fluorescent non-hydrolysable glucose analogue, as described previously [36]. Cells from the early exponential phase of growth were washed and suspended in sterile PBS with a pH of 7.4. 6-NBDG was added to the cell suspension at a final concentration of 150 μM and incubated for 90 min. at 28 °C. After incubation, the reaction was stopped by washing the cells twice with PBS. The fluorescence of the cells was recorded using a Tecan Infinite 200 microplate reader (Tecan Austria GmbH, Salzburg, Austria) at λex = 455 nm and λem = 540 nm. The quantitative results are presented as the mean value from three independent experiments.

### 2.13. Assessment of Relative Glucose Consumption

Relative glucose consumption was estimated by measuring the drop of glucose concentrations in the medium during a 4 h culture of cells with a synchronised output cell density (1 × 10^7^ cells/mL). Quantitative determination of glucose concentration in the culture medium after yeast cell removal was performed using the Amplex Red Glucose/Glucose Oxidase Assay Kit (Molecular Probes, Eugene, OR, USA) according to the manufacturer’s protocol. Fluorescence was measured after 20 min of incubation at 28 °C in the dark, using a Tecan Infinite 200 microplate reader (Tecan Group Ltd., Männedorf, Switzerland) at λex = 530 nm and λem = 590 nm. Glucose content was calculated from a standard curve. Glucose consumption was determined by the difference in glucose content in the medium between time 0 and 4 h of culture. The values are expressed as arbitrary units relative to the glucose consumption of WT strain cells cultured in 2% glucose medium.

### 2.14. Statistical Analysis

The results are presented as mean ± SD from at least three independent experiments (apart from the determination when individual cells were analysed). The statistical analysis was performed using STATISTICA 13.3 software (StatSoft, Inc., Krakow, Poland). The statistical significance of the difference between values obtained in media containing 2% and 0.5% glucose was evaluated using a *t*-test for independent samples. The statistical significance of differences between yeast strains relative to the WT strain was assessed using a one-way ANOVA with the Tukey post hoc test. Homogeneity of variance was assessed using Levene’s test. The values were considered significant at *p* < 0.05. Used designation: differences in comparison to WT strain culture on defined medium * *p* < 0.05, ** *p* < 0.01, *** *p* < 0.001; differences between media with 2% and 0.5% glucose # *p* < 0.05, ## *p* < 0.01, ### *p* < 0.001. The used designation is explained in the legend of the figure it concerns.

## 3. Results

### 3.1. Deletion of RP Genes Differentially Slows Cell Population Growth

**Figure 1:**
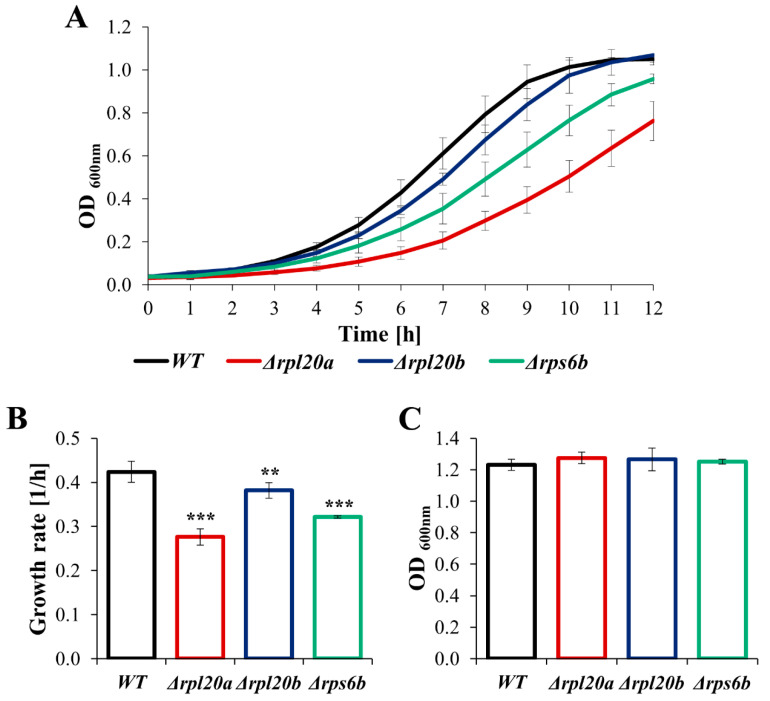
The growth of WT and RPs mutant yeast strains cultured in standard YPD medium (**A**) Growth kinetics of yeast cell population monitored during 12 h, (**B**) The growth rate of the yeast cell population, (**C**) The growth of the yeast cell population monitored after 24 h. The results are presented as mean ± SD from three independent experiments. ** *p* < 0.01; *** *p* < 0.001 as compared to WT strain.

In previous studies evaluating the effects of RP gene deletions, the most common phenotypes were growth defects and decreased growth rates [3,27,37]. Deletion of *RPL20A*, *RPL20B*, and *RPS6B* genes also resulted in slower growth of the cell population (Figure 1), although the growth rates of individual strains differed significantly. The differences in comparison to the growth of the WT strain were especially noticeable for strains lacking RPs paralogs, i.e., Δ*rpl20a* and Δ*rpl20b* (Figure 1A,B). The growth rate of the Δ*rpl20b* strain was only slightly reduced, while the growth of the Δ*rpl20a* strain was significantly decreased (Figure 1A), and the specified growth rate of this strain was one-third lower than the growth rate of the WT strain (Figure 1B). Nonetheless, similar growth of the WT and RPs mutant strains was observed after 24 h of cultivation (Figure 1C), indicating that the lower growth rate of the RPs mutant strains was due to less efficient biosynthesis rather than a blockage of cellular replication.

### 3.2. Deletion of RP Genes Reduces Cell Size and Increases Generation Time to Varying Degrees

**Figure 2:**
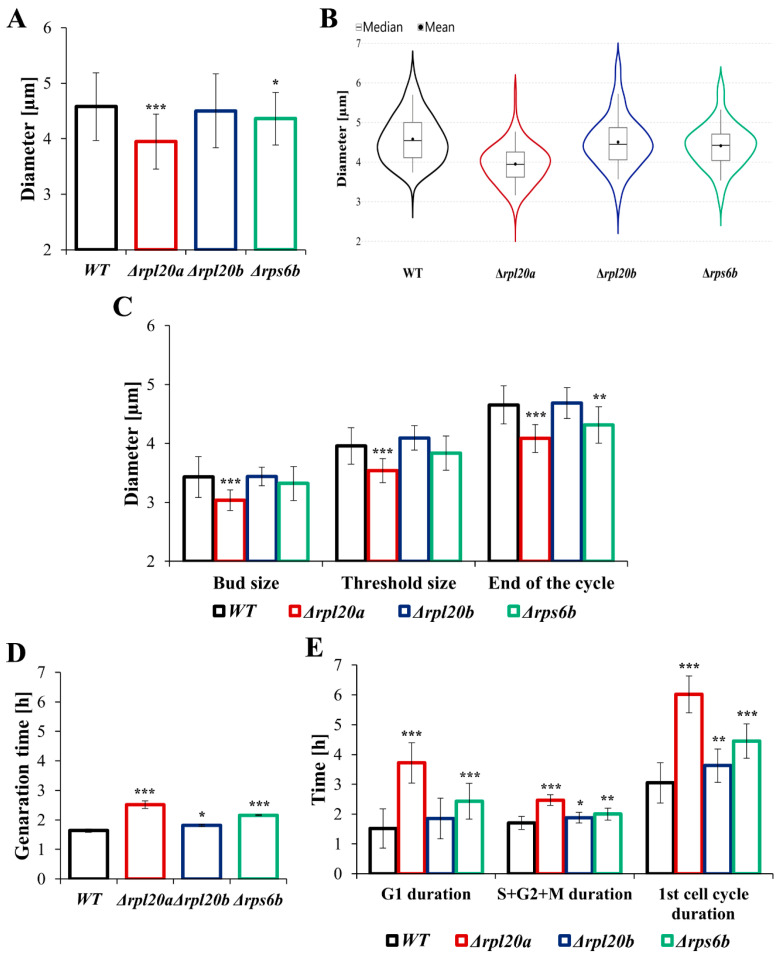
The cell size, generation time, and duration of the cell cycle determined in a cell population and in individual cells during the first cell cycle. (**A**) Mean cell size of WT and RPs mutant yeast strains measured in the population. The diameter of the cells was estimated through analysis of microscopic images using the cellSens Dimension software; n ≥ 200 cells, (**B**) Violin plots, a graphic representation of the distribution of cell diameter in the population of cells, (**C**) The cell size of WT and RPs mutant yeast strains measured during 1st cell cycle. Cell diameters were measured during micromanipulation. Analysis of microscopic images was performed using the Micro Image 3.0 programme, (**D**) Mean generation time of cells in the population calculated from the exponential phase of growth, (**E**) The generation time and duration of particular cell cycle phases of WT and RPs mutant yeast strains measured during 1st cell cycle. The results are presented as mean ± SD from two/three independent experiments. * *p* < 0.05; ** *p* < 0.01; *** *p* < 0.001 as compared to WT strain.

Maintaining cellular homeostasis requires the proper amount of cell components necessary for cell growth and division/reproduction. Therefore, in proliferating populations, cell size reflects a balance between cell growth and reproduction [38]. To investigate the effect of deletions of a given ribosomal protein on cell size and generation time, these parameters were measured both for the population of cells in the exponential growth phase and during the 1st cell cycle of buds for which this is their first cell cycle, marking the start of their reproductive stage. What follows from the fact that 1st cell cycle is the most demanding stage of cell life in terms of biosynthesis, particularly due to threshold size requirements [39,40]. Compared to the WT strain, mean cell size in the population was significantly decreased in the Δ*rpl20a* strain, only slightly decreased in the Δ*rps6b* strain, and not changed in the case of Δ*rpl20b* (Figure 2A). At the same time, cells exhibited relatively high variability in size (Figure 2A), which primarily arises from asymmetry in yeast cell division/budding, but can also be linked to variability in cell growth rate, cell size, or cell-cycle length. The distribution of cell diameter in the analysed population, presented in violin plots, showed significant differences between WT and Δ*rpl20a* strains, as well as between WT and Δ*rps6b*. In contrast, the cell diameter distribution of the Δ*rpl20b* strain is similar to that of the WT strain (Figure 2B). The growth rate (Figure 1B) and cell size in the population (Figure 2A) inversely correlate with the generation time determined from the growth curve (Figure 2D). The longest generation time was noted for Δ*rpl20a*, whereas the generation time of the WT strain and Δ*rpl20b* strain was more or less similar (Figure 2D). The results demonstrated that cells of the Δ*rpl20a* strain have the lowest biosynthetic capabilities and require more time to acquire the cellular equipment needed for proliferation. On the other hand, deleting its paralog, *RPL20B*, has a negligible effect on the cell’s biosynthetic efficiency. Differences in cell size between strains were also observed at specific stages of the 1st cell cycle (Figure 2C). The diameter of separated buds (virgin cells) was similar between strains; only smaller buds were noticed for the Δ*rpl20a* strain. As the cell cycle progressed and the cells grew, the differences in their size became more pronounced, and by the end of the first cell cycle, the cell size of only the Δ*rpl20b* strain was comparable to that of the WT strain. The cells of Δ*rpl20a* and Δ*rps6b* strains were smaller than the WT strain (Figure 2C), and the differences were more pronounced than those observed in the case of the whole cell population (Figure 2A). The reduced biosynthetic efficiency of the Δ*rpl20a* and Δ*rps6b* strains resulted in a significantly prolonged cell cycle (Figure 2E). The 1st cell cycle, which is already longer than the rest of the cell cycles during its life, was generally increased in all strains with RP gene deletion. The length of this cell cycle was doubled in the case of the Δ*rpl20a* strain and 50% greater in the case of the Δ*rps6b* strain compared to the WT strain. It is worth noting that this tremendous increase in cell cycle length primarily results from a significant prolongation of the G1 phase, i.e., the period during which cellular component biosynthesis occurs most intensively. A particularly long G1 phase (longer than the duration of the whole 1st cell cycle in the WT strain) was noted for cells of the Δ*rpl20a* strain. The differences in time spent in the sum of S, G2, and M phases were unquestionable but less important for the length of the entire cell cycle (Figure 2E). The results indicate that smaller cells with lower biosynthetic capabilities may exhibit a more extended G1 phase.

### 3.3. Deletion of RP Genes Reduces Cell Size Growth During the Cell Cycle Arrest in the G1 Phase

**Figure 3:**
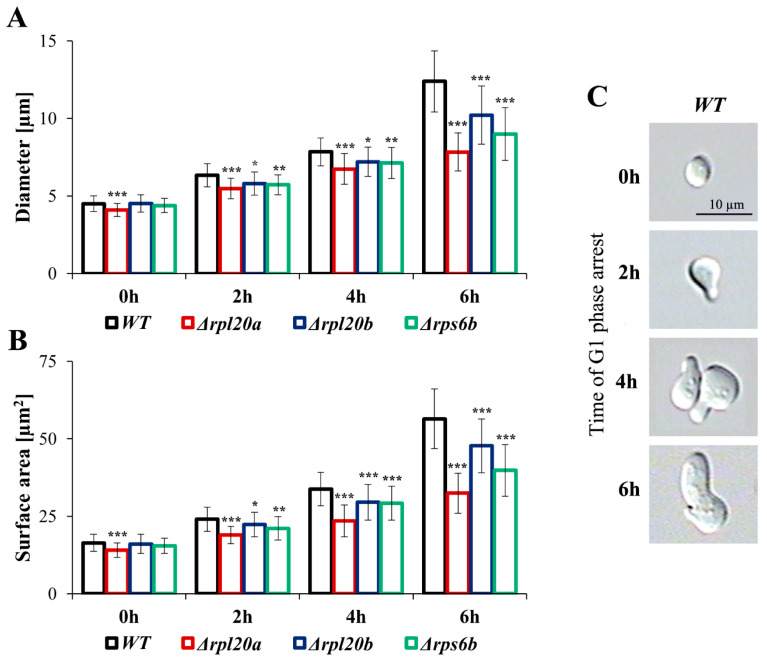
Changes in cell size of WT and RPs mutant yeast strains during the cell cycle arrest in the G1 phase. (**A**) Mean cell size measured as a diameter of the cells after the specified time with pheromone, (**B**) Mean cell size measured as a surface area of the cells after the specified time with pheromone, (**C**) Visualisation of changes in the shape of the cells during incubation with pheromone. Diameter and surface area were estimated from microscopic images using the cellSens Dimension software; n ≥ 100 cells. The results are presented as mean ± SD from two independent experiments. * *p* < 0.05; ** *p* < 0.01; *** *p* < 0.001 as compared to WT strain.

In proliferating cells, the cellular resources must be allocated between cell maintenance and reproduction. The separation of these two closely connected bioprocesses enables a more detailed analysis of the cell’s biosynthetic efficiency. To decouple biosynthetic productivity from reproduction and population growth in *Saccharomyces cerevisiae*, pheromone treatment can be employed, as it induces cell cycle arrest in the G1 phase without inhibiting cell biosynthesis or growth [41]. Changes in cell size during pheromone-induced cell-cycle arrest (measured using both cell diameter and cell area due to irregular shapes) in strains with RP gene deletions were analysed (Figure 3A–C). Cell size increased significantly during the time of cell cycle arrest in the G1 phase (Figure 3A,B). For both parameters, the cell size of the WT and strains with deletion of the RPs gene (except for Δ*rpl20a*) was similar at the beginning; however, as the time of cell cycle arrest increased, the disparity in cell size increased. After 6 h of cell cycle arrest, the smallest cell size and the lowest increase in cell size growth in comparison to the WT strain were observable for Δ*rpl20a*. In turn, changes in cell size in the Δ*rpl20b* strain were most similar to those observed in the WT strain (Figure 3A,B). The obtained results confirm the dependencies between cell size and the biosynthetic capabilities of cells with the deletion of selected RP genes. They also show that differences in biosynthesis efficiency between them are greater when cells are released from reproductive pressure.

### 3.4. Deletion of RP Genes Reduces the Rate of Individual Cell Size Growth

**Figure 4:**
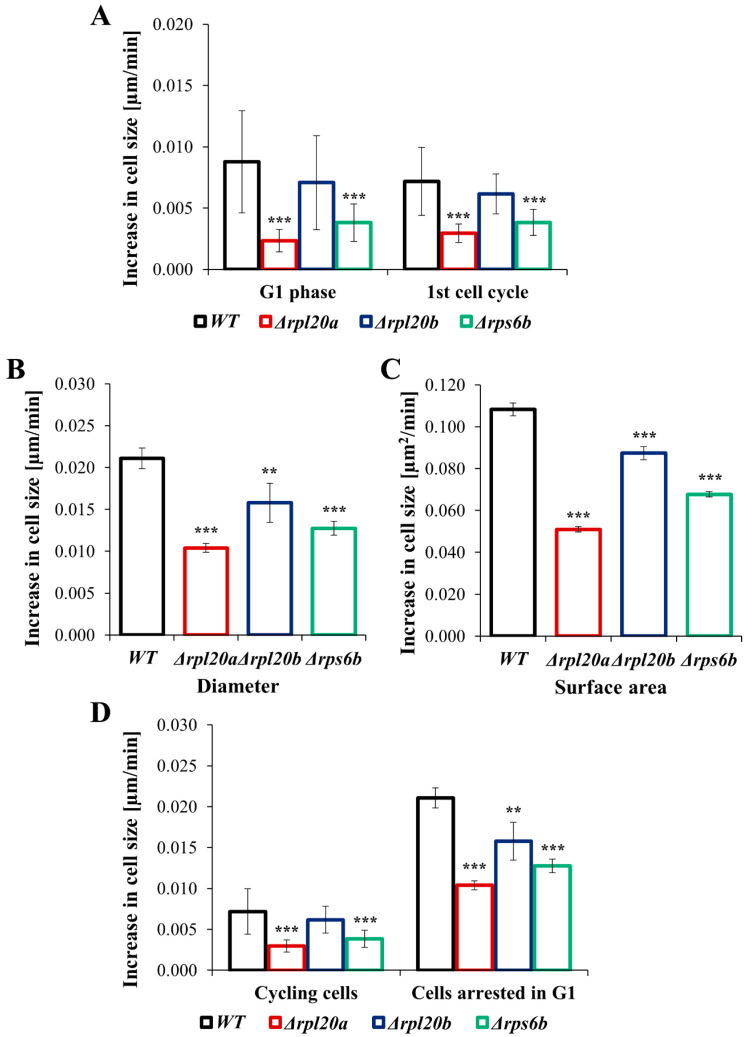
The rate of increase in cell size of WT and RPs mutant yeast strains during the first cell cycle and the cell cycle arrest in the G1 phase. (**A**) Increase in cell size measured during 1st cell cycle, (**B**) Increase in cell size measured as changes in diameter after the specified time with pheromone, (**C**) Increase in cell size measured as changes in surface area after the specified time with pheromone, (**D**) Comparison of the rate of increase in cell size in cycling cells and cells arrested in G1 phase. The results are presented as mean ± SD from two independent experiments. ** *p* < 0.01; *** *p* < 0.001 as compared to WT strain.

To compare the growth rate and biosynthetic capabilities of individual cells, cell size was measured over a defined time period. Both proliferating cells and cells arrested in the cell cycle were analysed. The increase in cell size during the 1st cell cycle was relatively variable between individual cells (Figure 4A); however, as in previous analyses, the Δ*rpl20a* strain showed the lowest rate of increase in cell size (about 5 times lower than the WT strain).

The lower increase in cell size was also noted for the Δ*rps6b* strain, whereas an increase in cell size in the Δ*rpl20b* strain was similar to the values pointed out for the WT strain (Figure 4A). A similar tendency toward increased cell size across strains was observed in cells arrested in the G1 phase (Figure 4B,C). The lowest increase in cell size was observed for the Δ*rpl20a* strain, and the increase in cell size was also lowered in the Δ*rps6b* strain; under these conditions, the Δ*rpl20b* strain showed a slight reduction in the increase in cell size (Figure 4B,C). The comparison of the increase in cell size between cycling cells and cells arrested in the cell cycle (Figure 4D) showed that the rate of cell growth is much higher (about 4 times) in cells arrested in the G1 phase. However, the dependencies between strains are maintained, as indicated by the substantial degree of correlation between the results (Pearson’s r = 0.86; *p* < 0.00).

### 3.5. Deletion of RP Genes Changes the Energetic and Biosynthetic Capacities of the Cells to Varying Degrees but Does Not Induce Higher Mortality or Decreased Overall Vitality

**Figure 5:**
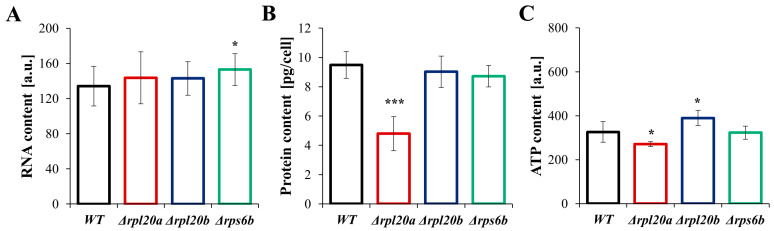
Parameters connected with energetic and biosynthetic capacities in WT and RPs mutant yeast strains (**A**) Relative RNA content of yeast cells assessed with acridine orange. Fluorescence was examined under the fluorescence microscope at λex = 488 nm and λem = 650 nm. The relative value of RNA was measured from red fluorescence after separating the multichannel images into individual colour channels, (**B**) Protein content in cell extracts obtained from a strictly defined number of cells (5 × 10^8^ yeast cells from the exponential phase culture) expressed in the pg per cell, (**C**) Intracellular ATP content in the yeast cells determined with BacTiter-Glo™ Microbial Cell Viability Assay. The results are presented as mean ± SD from two/three independent experiments. * *p* < 0.05; *** *p* < 0.001 as compared to WT strain.

**Figure 6:**
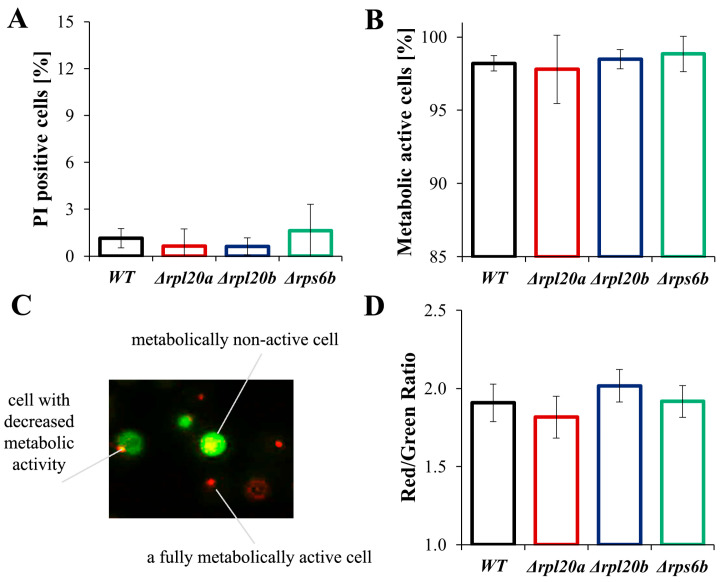
Vitality and viability of WT and RPs mutant yeast strains (**A**) Cell viability determined by staining with PI and FDA. FDA/PI fluorescence was examined with a fluorescence microscope at λex = 480 nm; n ≥ 100 cells for each repetition, (**B**) Metabolic activity of yeast cells, treated as an equivalent of cell vitality, was determined with FUN^®^1. Metabolically active and inactive cells were examined based on fluorescence images of cells captured with an Olympus BX-51 microscope equipped with the DP-72 digital camera; n ≥ 150 cells for each repetition, (**C**) visualisation of metabolic differences between yeast cells estimated by staining with FUN^®^1, (**D**) Differences in metabolic activity of cells expressed as a ratio of red (λ = 575 nm) to green (λ = 535 nm) fluorescence. The fluorescence of the cell suspension was measured using a TECAN Infinite 200 microplate reader at λex = 480 nm and λem = 500–650 nm. The results are presented as mean ± SD from three independent experiments. The values were considered significant at *p* < 0.05.

To gain insights into the physiological aspects of growth and cell size that change in strains with RP gene deletions, RNA, protein, and ATP content were examined (Figure 5A–C). It was also tested whether the observed differences influence cell viability and vitality (Figure 6A–C). The determined relative RNA content was similar across the analysed strains, with only a slightly higher level observed for Δ*rps6b* (Figure 5A). The estimated protein content (Figure 5B) corresponds to the previously determined growth and cell size parameters (Figure 1, Figure 2, Figure 3, Figure 4 and Figure 5). Cells with deletion of a particular RP gene tend to have lower protein content. However, statistical significance was noted only for the Δ*rpl20a* strain, in which protein content was almost 2 times lower than in the WT strain (Figure 5B). Notably, total RNA levels in the Δ*rpl20a* yeast strain were not decreased relative to cell size or protein content. Furthermore, in the Δ*rpl20a* strain, the reduction in total protein content was accompanied by a relatively high total RNA content, resulting in a markedly lowered protein/RNA ratio (Figure 5A,B). Cells of the Δ*rpl20a* strain also had lower ATP content (Figure 5C). The ATP content in the Δ*rps6b* strain was similar to values noted for the WT strain, whereas the ATP content in Δ*rpl20b* was a bit higher (Figure 5C).

The results confirm that the Δ*rpl20a* and Δ*rpl20b* strains differ significantly in biosynthetic efficiency. The Δ*rpl20a* strain shows a considerably reduced biosynthetic capacity, whereas biosynthesis in the Δ*rpl20b* strain is comparable to that of the WT strain. Despite differences in biosynthetic potential, no significant changes in viability (referring to the number of live cells within a population) or vitality (concerning the physiological capabilities of cells) were observed in strains with an RP gene deletion (Figure 6A–D). There was no increase in the number of dead cells analysed with FDA/PI staining (Figure 6A), and there were also no significant differences in cell vitality and percentage of active cells determined by fluorescence microscopy images (Figure 6B,C). Since active cells may vary in their degree of activity (Figure 6C), quantitative measurements of cell fluorescence and the Red/Green fluorescence ratio were performed. No statistically significant differences were also found in this type of determination (Figure 6D).

### 3.6. Deletion of RP Genes Decreases Glucose Consumption and the Rate of Glucose Uptake

**Figure 7:**
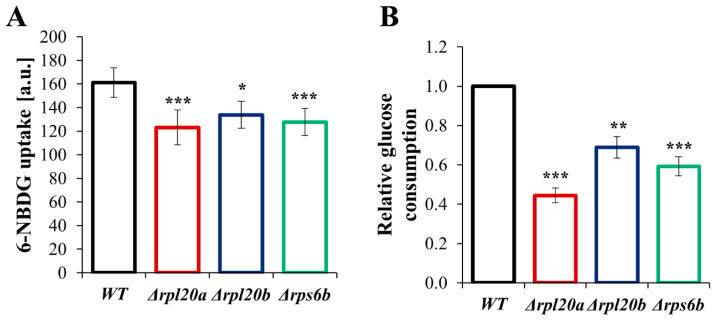
The glucose uptake in WT and RPs mutant yeast strains (**A**) The rate of glucose uptake by yeast cells of WT and RPs mutant yeast strains. The rate of glucose uptake was measured by measuring the fluorescent signal after incubation with 6-NBDG. (**B**) The relative glucose consumption of WT and RPs mutant yeast strains was determined by measuring the drop in glucose concentration in the medium after 4 h of culture at a synchronised output cell density. The results are presented as mean ± SD from three independent experiments. * *p* < 0.05; ** *p* < 0.01; *** *p* < 0.001 as compared to WT strain.

Since cellular metabolism is connected and adapted to the presence and uptake of carbohydrates in the medium, parameters related to glucose consumption were also tested (Figure 7A,B). The level of glucose uptake, measured with the fluorescent non-hydrolyzable glucose analogue 6-NBDG, was reduced in all RP-gene-deletion strains compared to the WT strain. The lowest glucose uptake was noted for the Δ*rpl20a* strain (Figure 7A). The same dependencies were observed for relative glucose consumption, as evidenced by a drop in medium glucose concentration (Figure 7B). In comparison to the WT strain, the glucose consumption was lower by more than 50% in the Δ*rpl20a* strain, about 40% in the Δ*rps6b* strain, and 25% in the case of the Δ*rpl20b* strain. The results indicate that lower biosynthetic capabilities resulting from RP gene deletion reduce the carbohydrate consumption rate.

### 3.7. Calorie Restriction Does Not Decrease Biosynthesis in Strains with Initially Low Biosynthetic Efficiency

**Figure 8:**
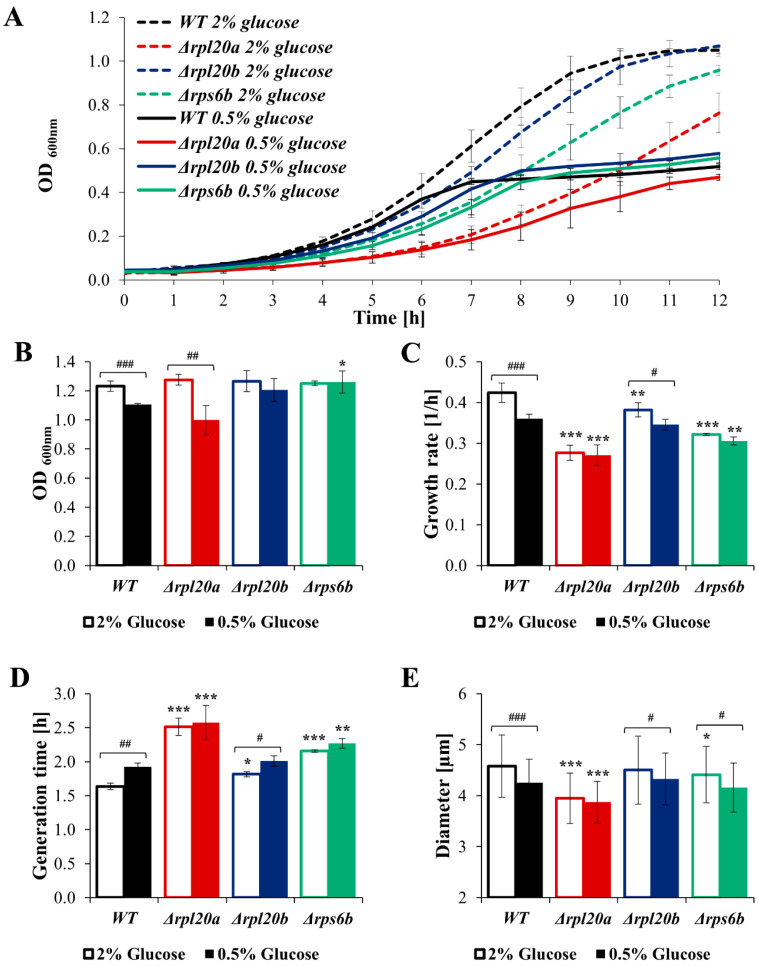
The cell size, generation time, and growth of WT and RPs mutant yeast strains cultured in conditions with different glucose concentrations (2% glucose—optimal conditions, open boxes; 0.5%—CR conditions, filled boxes; this applies to all colours). (**A**) Growth kinetics of yeast cell population monitored during 12 h of culture in standard YPD medium and in CR conditions. (**B**) The growth of the yeast cell population monitored after 24 h of culture in optimal and CR conditions. (**C**) The growth rate of the yeast cell population in optimal and CR conditions. (**D**) Mean generation time of cells in optimal and CR conditions calculated from the exponential phase of growth. (**E**) Mean cell size of WT and RPs mutant yeast strains cultured in optimal and CR conditions. The cell diameter was estimated from microscopic images using the cellSens Dimension software (n ≥ 200 cells). The results are presented as mean ± SD from two/three independent experiments. * *p* < 0.05; ** *p* < 0.01; *** *p* < 0.001 as compared to WT strain; # *p* < 0.05; ## *p* < 0.01; ### *p* < 0.001 as compared between optimal and CR conditions.

**Figure 9:**
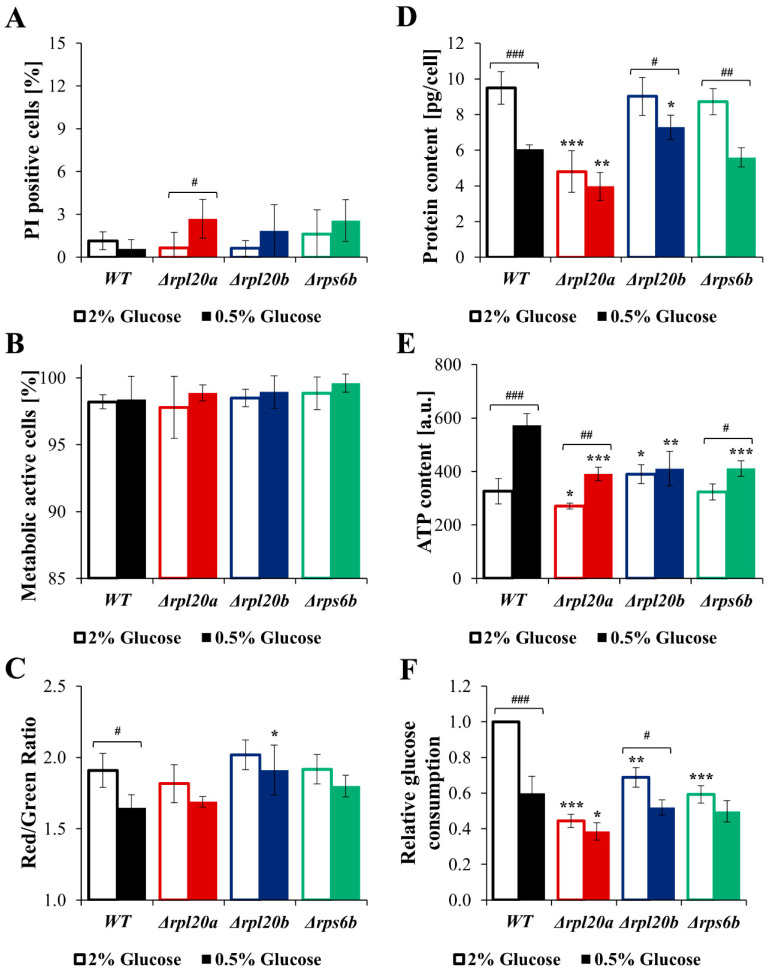
Parameters connected with vitality, energetic and biosynthetic capacities, and glucose consumption in WT and RPs mutant yeast strains cultured in conditions with different glucose concentrations (2% glucose—optimal conditions, open boxes; 0.5%—CR conditions, filled boxes; this applies to all colours). (**A**) Cell viability determined by staining with PI and FDA. FDA/PI fluorescence was examined with a fluorescence microscope at λex = 480 nm; n ≥ 100 cells for each repetition. (**B**) Metabolically active yeast cells determined with FUN^®^1. Metabolically active and inactive cells were examined based on fluorescence images of cells captured with an Olympus BX-51 microscope equipped with the DP-72 digital camera; n ≥ 150 cells for each repetition. (**C**) Differences in metabolic activity of cells expressed as a ratio of red (λ = 575 nm) to green (λ = 535 nm) fluorescence. (**D**) Protein content in cell extracts obtained from a strictly defined number of cells (5 × 10^8^ yeast cells from the exponential phase culture) expressed in the pg per cell. (**E**) Intracellular ATP content in the yeast cells determined with BacTiter-Glo™ Microbial Cell Viability Assay. (**F**) The relative glucose consumption of WT and RPs mutant yeast strains determined by measuring the drop of glucose concentrations in the medium after 4 h culture with a synchronised output cell density. The results are presented as mean ± SD from two/three independent experiments. * *p* < 0.05; ** *p* < 0.01; *** *p* < 0.001 as compared to WT strain; # *p* < 0.05; ## *p* < 0.01; ### *p* < 0.001 as compared between optimal and CR conditions.

As cell size and biosynthesis efficiency are linked to glucose consumption rate, the question arises whether different nutrient conditions may further modulate cellular biosynthesis. Therefore, the analysis of growth, cell size, viability, and biosynthetic activity of strains with RP gene deletions under CR conditions, the best-known nutrient alteration that lowers biosynthesis and simultaneously increases lifespan and improves cell and organism physiology [36,42,43,44], was performed (Figure 8A–E and Figure 9A–F). Compared with the medium containing 2% glucose, the growth of the cell population in the medium containing 0.5% glucose was reduced, though to varying degrees depending on the strain. The kinetic growth curves in CR conditions showed greater similarity between strains with RP gene deletions and the WT strain than those at 2% glucose (Figure 8A). A clear difference in growth was pointed out in the case of the Δ*rpl20a* strain (Figure 8A). Cell growth in CR conditions after 24 h of cultivation was similar among the analysed yeast strains. However, in WT and Δ*rpl20a* strains, the OD values after 24 h were lower in medium containing 0.5% glucose than in medium containing 2% glucose (Figure 8B). The difference between standard (2% glucose) and CR conditions in the specified growth rate (Figure 8C) was observed in strains with initially high growth rates, i.e., WT and Δ*rpl20b*. The growth rate in the Δ*rpl20a* and Δ*rps6b* strains does not decrease under CR conditions compared to medium containing 2% glucose (Figure 8C). Inversely, the generation time increased under the CR condition compared with the standard condition, but only in WT and Δ*rpl20b* strains (Figure 8D). There were no statistically significant changes in generation time between conditions in the case of Δ*rpl20a* and Δ*rps6b* strains. However, the Δ*rpl20a* strain still had a significantly longer generation time than other strains (Figure 8D). The mean cell size in the population under standard and CR conditions decreased substantially in all analysed strains, except the Δ*rpl20a* strain, which remained smaller than that of the other strains (Figure 8E). The CR conditions did not significantly affect cell viability and vitality (Figure 9A–C). A slightly higher degree of cell mortality was noted under CR conditions in the case of the Δ*rpl20a* strain (Figure 9A). There were no changes in the percentage of metabolically active cells (Figure 9B). However, under CR conditions, cells of all strains showed a tendency to decrease their activity, especially in the WT strain (Figure 9C). The changes in energetic and biosynthetic capacities of cells between standard and CR conditions (Figure 9D–E) depend on the initial values. The decrease in protein content under CR conditions was more pronounced for strains with higher initial biosynthesis efficiency. The highest drop in protein content was noted for the WT strain, whereas the protein content in the Δ*rpl20a* strain between standard and CR conditions was almost unchanged (Figure 9D). In contrast to protein content, ATP content increased between standard and CR conditions (Figure 9E). The highest values and most pronounced increase in ATP content were noted in the WT strain, whereas the increase in ATP content in Δ*rpl20a* and Δ*rps6b* strains was modest (Figure 9E). In the case of the Δ*rpl20b* strain, a slight decrease in protein content (Figure 9D) and an almost unchanged ATP content (Figure 9E) were observed between standard and CR conditions. The analysis of relative glucose consumption (Figure 9F) completes the picture of changes under CR conditions. Glucose consumption decreased significantly in both WT and Δ*rpl20b* strains. In contrast, it was non-reduced or only slightly reduced in the strains that initially had lower glucose uptake rates, i.e., the Δ*rpl20a* and Δ*rps6b* strains (Figure 9F). The results confirm that one impact of CR on cells is a reduction in their biosynthetic capabilities. However, analysis using strains with a specific RP gene deletion indicates that CR impact does not occur in strains with initially low biosynthetic efficiency. This suggests that genetic and environmental approaches reducing cellular biosynthesis do not appear to have a synergistic effect.

## 4. Discussion

### 4.1. Deletions of Ribosomal Genes and Their Phenotypic Aspects

Biosynthetic processes, especially those involved in protein synthesis, are essential for cell growth and proliferation. Several studies indicate that enhancing protein turnover, which can be achieved by reducing protein biosynthesis, positively influences cell viability and proliferative capacity. Therefore, in this work, we analysed the impact of deletions in selected RP genes on cell physiological parameters, both in standard (2% glucose) complete medium and in CR conditions (0.5% glucose). To draw broader conclusions and assess the possibility of functional specificity of ribosomal proteins, we used strains lacking RPs from the large and small subunits, including strains with RP paralogs deletion. As previously noted [4,45], the most common phenotypes associated with RP gene deletion are decreased growth rate (Figure 1), reduced cell size, delayed cell proliferation (Figure 2 and Figure 3), and reduced protein synthesis (Figure 5). However, deleting specific RP genes results in distinct phenotypic profiles, as we showed in the case of the RP mutant strains used: phenotypes similar to the wild strain (Δ*rpl20b*), slightly reduced growth (Δ*rps6b*), and significantly reduced growth and biosynthetic potential (Δ*rpl20a*). It is worth emphasising that the most divergent phenotypes were observed in the Δ*rpl20a* and Δ*rpl20b* strains, which are paralogs, thereby confirming the possibility of a paralog-specific role for ribosomal proteins [46,47]. Several mechanisms have been proposed to explain the phenotypic diversity of paralogs, such as: (i) gene dosage model and differences in RP paralogs expression [17,46,47,48]; (ii) differences in protein levels and imbalances in ribosomal subunits among individual paralog forms [17,37]; (iii) existence of a ‘ribosomal code’, whereby different RP isoforms are incorporated into heterogeneous ribosomes that selectively translate a specific type of mRNA [46]; or (iv) extra-ribosomal function of RPs [49]. A combination of these mechanisms can lead to a paralog-specific phenotype, as seen in the Δ*rpl20a* strain. The notably low protein/RNA ratio in the Δ*rpl20a* strain may suggest a significant disruption in ribosome assembly or maturation. Blocking ribosome assembly could cause the accumulation of immature rRNA forms. Research indicates that blocking 60S assembly by deleting ribosomal proteins leads to an accumulation of intermediate pre-rRNA forms, such as 35S, 27S, and 7S, which may increase rRNA and total RNA content despite a decrease in protein synthesis [50,51,52,53]. This may explain why total RNA does not decrease in proportion to cell mass or protein content (Figure 2, Figure 4 and Figure 5). Furthermore, Yeah and Lee have shown that the yeast 60S subunit protein L20 interacts specifically with 35S pre-RNA [54], thereby influencing assembly and, consequently, ribosome biogenesis. Taking into account the literature data and our results, it seems that, of the pair of paralogues Rpl20a/Rpl20b, Rpl20a plays a crucial role in ribosome assembly due to its interaction with pre-RNA [54]. This interpretation aligns with the compensatory genomic responses reported for *RPL20A* deletion, such as the spontaneous duplication of the *RPL20B* locus in rapidly growing revertants. It further suggests that the underlying defect affects ribosome function rather than merely RNA production. Such a possibility was previously noted in the slowly growing Δ*rpl20a* strain [55,56] and was also confirmed by the appearance of fast-growing derivatives in the Δ*rpl20a* strain after a longer culture time. In the present work, it was also found that strains with the deletion of RP paralogs significantly differ in duration of the G1 phase (Figure 2); increasing in cell size measured both in cycling cells and cells arrested in G1 with pheromone (Figure 3 and Figure 4); glucose uptake rate and energetic capabilities of the cells (Figure 5 and Figure 7). In all parameters analysed, deletion of the *RPL20A* gene severely impaired the cells’ physiological efficiency. This provides further evidence confirming the crucial role of the Rpl20a protein in maintaining proper ribosomal function, despite the presence of a paralog, Rpl20b, with 99% nucleotide identity.

### 4.2. The Relationships Between Physiological Efficiency, Cell Size, and Proliferation

Considering proliferation as a measurable aspect of a cell’s physiological efficiency, important parameters derived from the cell’s biosynthetic potential are the time of a single reproductive cycle and cell size [57,58]. During each cell cycle, cells must synthesise several proteins, including those responsible for crossing subsequent phases of the cell cycle, as well as proteins and enzymes related to the proper functioning and growth of the proliferating cell. The first cell cycle is significantly longer than the others because the cell must reach a critical size, and its length primarily depends on the size of the separated buds [59] and the rate of cell growth. Hence, small cell sizes may result from a mutation that reduces growth rate and is generally associated with modulation of respiration and ribosome biogenesis [60,61,62]. Additionally, mutations that significantly reduce growth rate lengthen the cell cycle, mainly by extending the G1 phase [63]. In this work, it was also noted that deletion of RP genes affects cell growth rate and cell cycle length, particularly the G1 phase (Figure 2 and Figure 4). An exceptionally prolonged cell cycle and G1 phase characterise cells of the Δ*rpl20a* strain (Figure 2), and the twice-longer cell cycle in the Δ*rpl20a* strain compared to the wild strain has been reported previously [55,63]. Moreover, the lower growth rate and the more extended G1 phase and cell cycle duration are directly associated with smaller cell sizes (Δ*rpl20a* and Δ*rps6b*). In their case, the extended G1 phase is intended to compensate for a smaller cell size [39]. Nevertheless, such compensation is not always sufficient, as observed in the Δ*rpl20a* strain, in which cells begin budding at smaller cell sizes, reducing the so-called threshold size (Figure 2C).

*Saccharomyces cerevisiae* yeast cells cultured under optimal conditions continue to bud until they deplete their nutrients, although many other factors also influence cell proliferation. One of them is undoubtedly the cell size, since proliferating cells must coordinate cell-cycle progression with their growth and biosynthetic possibilities [38,40,64]. The concept of a threshold size—the dimension each cell must reach to start budding—is widely accepted and, in budding yeast, is controlled at the G1/S transition at the “Start” point [39,40,65]. The existence of this size is, on the one hand, necessary to prevent cells from gradually shrinking. Still, on the other hand, it also explains differences in cycle length between cells of different initial sizes. Moreover, the threshold size appears to be tightly connected to the size-sensing mechanism based on the synthesis rate [40,66,67].

Changes in cell size across subsequent reproductive cycles are also crucial; the increase in size during each cycle makes cells too large, inhibiting their further proliferation. This relates directly to the proposed hypertrophy hypothesis, which was put forward more than a decade ago, as an alternative explanation for the limited proliferative capacity of cells [68,69]. Recently, more studies have investigated the relationships among cell size, biosynthesis rate, cellular physiology, and cell reproductive potential [27,43,58,66,70]. To explore whether and how cells maintain optimal function with cell size, different approaches using chemical compounds (e.g., rapamycin, cycloheximide, tunicamycin), genetic (ploidy or deletion of selected genes), and environmental (nitrogen and carbon availability) changes are used [35,43,45,66]. Undeniably, maintaining the physiological efficiency of the cell requires scaling the metabolism, biosynthesis, and subcellular structures with cell size and environmental conditions [71]. Improper regulation of physiological processes that scale with cell size has recently been linked to reduced proliferation capacity and premature senescence in both yeast and mammalian cells [70,72]. The size of the cell is directly dependent on the levels and functional efficiency of proteins, which are strictly related to ribosome biogenesis and function. In this context, most works analysed the influence of ribosomal protein (RP) gene deletions [27,45,73].

Changes in cell size observed in proliferating cells are tightly controlled by factors regulating the cell cycle. Therefore, to more fully assess the impact of a cell’s biosynthetic potential, the rate of cell size increase during G1 arrest was measured. This gives a much more precise picture of the effects of biosynthetic capabilities on cell size and the rate of change in this parameter over time. This experimental approach confirmed an approximately two-fold lower rate of cell size increase in the Δ*rpl20a* strain and significantly lower rates of cell size increase in the Δ*rps6b* and Δ*rpl20b* strains. As shown by statistical analysis, increases in cell size during the 1st cell cycle and during G1 arrest are strongly positively correlated. However, the increase in cell size in cycling cells is significantly lower than the increase in cell size when cells are arrested in the G1 phase (Figure 2, Figure 3 and Figure 4). This result suggests that the low rate of cell size increase may be a key mechanism underlying higher cell proliferation activity.

### 4.3. Calorie Restriction and Deletion of Ribosomal Genes in Reducing Cellular Biosynthesis

Deletion of RP genes leads to a measurable decline in biosynthetic capacity, related to reduced nutrient utilisation. Consistent with this, analysed RP-deletion strains—particularly Δ*rpl20a*—exhibited lowered glucose uptake (Figure 7), indicating that impaired biosynthesis may influence cell energy metabolism and overall cellular physiological status. Although slight differences in ATP levels were observed compared to the WT strain, deletion of the analysed genes did not affect cell viability or significantly alter cell vitality (Figure 5 and Figure 6). This observation may be indirect evidence that the reduction in biosynthesis has no negative impact, and, conversely, may help explain the prolonged lifespan observed in mutant strains associated with the deletion of RP genes and ribosome biogenesis [27,62,73,74]. Calorie restriction is another intervention known to extend lifespan and improve cellular health span by lowering metabolic activity and overall cellular biosynthetic [43,44,75]. The mechanism linking deletions of RP genes, CR, and reductions in cellular biosynthesis to extending lifespan appears to involve maintenance of the protein quality control system, which in yeast cells can be modulated by nutrient availability [44,58,60]. Hence, a general decrease in biosynthesis under CR conditions may improve cellular physiology by better control of the synthesis, modification, repair, and degradation of proteins [43,44,58,76,77]. Taking this into account and recognising that deletion of RP genes reduces glucose uptake, we asked what would happen when both approaches to reducing cellular biosynthesis are used. The results confirm that CR reduces the cells’ biosynthetic capabilities. However, the impact of CR does not occur in cells with initially low biosynthetic efficiency, as demonstrated by analysis of Δ*rpl20a* and Δ*rps6b* strains under CR conditions (Figure 8 and Figure 9). The growth rate, cell size, protein content, cell metabolic activity, and glucose consumption significantly decrease in the WT strain under CR conditions (Figure 8 and Figure 9). On the other hand, a negligible effect of CR was observed in the Δ*rpl20a* strain, in which a significant reduction in biosynthetic efficiency was already observed under optimal conditions. It is worth noting that the level of biosynthetic processes that ensures the appropriate level of macromolecules, such as proteins, DNA, and RNA, adapts to changes in cell size, allowing for the maintenance of physiological cell efficiency [67]. However, this occurs only to a certain extent, and reaching an excessive size disrupts this process, leading to cytoplasmic dilution [70]. Based on the results presented, it can be assumed that cell biosynthetic adjustment may also apply to minimal levels, which can be regarded as a threshold for maintaining physiological activity. Therefore, factors that decrease biosynthetic efficiency only have a noticeable effect on cells whose levels surpass this threshold. The existence of this threshold biosynthetic level may offer specific protection against loss of physiological efficiency caused by insufficient biosynthetic capacity. Although the particular standardisation of the measurable value of the biosynthetic threshold level is limited, its existence appears justified. This is indicated by observations of strains with ribosome-related gene deletions that show no further decrease in protein levels, no extension of generation time, and no reduction in growth rate in CR conditions, in contrast with strains with undisturbed biosynthesis. Data on the effects of ribosome-related gene deletions on the ER stress response and hypoxia tolerance may also support this hypothesis. In the cases cited, deleting genes involved in ribosome biogenesis reduces protein synthesis and growth rate, thereby protecting cells against ER stress and hypoxia-induced changes [45,78]. Therefore, cells cannot reduce their biosynthetic efficiency below the minimum threshold.

## 5. Conclusions

The results of this study provide new insights into the relationships between biosynthetic efficiency, cell size, and physiological efficiency, as reflected in the cell’s ability to proliferate. Reducing biosynthesis levels, mainly indicated by protein levels, does not decrease the cell’s physiological efficiency; instead, it enhances the efficient use of available substrates for biosynthetic processes. Additionally, the significant effect of lowering biosynthesis on the average cell size within the population, the threshold size needed to trigger the first cycle, and the rate of cell size growth during subsequent cycles strongly suggest that this could be a key factor in explaining how reducing biosynthesis promotes cell proliferation and lifespan. Moreover, these findings may help clarify the varied effects of CR. The beneficial effects of CR from reduced biosynthetic activity may depend on the initial activity level and the presence of a threshold that serves as a safeguard to preserve the cell’s physiological efficiency.

## Data Availability

The data generated and analysed during this study are available from the corresponding author upon reasonable request.
